# Research Trends and Hot Topics in Nursing Research on Children with Disabilities: A Bibliometric Analysis from 1977 to 2026

**DOI:** 10.3390/healthcare14172824

**Published:** 2026-09-03

**Authors:** Habibe Ozcelik, Şule Şenol, Hasan Huseyin Avci

**Affiliations:** 1 Department of Public Health Nursing, Faculty of Nursing, Akdeniz University, Antalya 07058, Türkiye; hozcelik@akdeniz.edu.tr; 2 Department of Pediatric Nursing, Faculty of Nursing, Akdeniz University, Antalya 07058, Türkiye; 3 Department of Family Medicine, Faculty of Medicine, Akdeniz University, Antalya 07070, Türkiye; hasanavci@akdeniz.edu.tr

**Keywords:** bibliometric analysis, children with disabilities, nursing, pediatric nursing, research trends, thematic evolution, science mapping

## Abstract

**Background/Objectives**: Nursing research on children with disabilities spans diverse disability groups, care settings, and areas of practice; however, its development and structure have not been comprehensively examined. This study aimed to examine publication trends, major research themes, temporal development, and collaboration patterns. **Methods**: The Web of Science Core Collection was searched on 29 June 2026 without publication-year restrictions. English-language articles and reviews indexed in Science Citation Index Expanded (SCI-EXPANDED) and Social Sciences Citation Index (SSCI) were included, yielding 1915 publications from 1977 to 2026. VOSviewer and Biblioshiny were used to analyze publication trends, keyword co-occurrence, thematic structure and evolution, trend topics, and country and institutional collaboration. **Results**: Research output increased substantially, particularly during the last decade. Across the study period, the United States had the highest publication output and co-authorship connectivity, followed by England, Canada, and Australia. Nursing, children, autism spectrum disorder, intellectual disability, and cerebral palsy were among the largest nodes in the keyword network. The thematic map positioned the autism–pediatrics–developmental disability cluster slightly within the motor themes quadrant, while the disability–children with disabilities–qualitative research, adolescents–communication–transition, and children–nursing–intellectual disability clusters were positioned among the basic themes. Thematic evolution showed both continuity and diversification, with autism, nursing, children, and intellectual disability represented across multiple periods, while quality of life, education, and mental health were represented in the most recent period. Trend topic analysis further showed that well-being, implementation, pediatric nursing, anxiety, and mental health were among the topics with more recent median publication years. **Conclusions**: Nursing research on children with disabilities has expanded and diversified, with recurring disability-specific topics alongside more recent topics related to psychosocial issues, pediatric nursing, and implementation. Future research could build on the thematic patterns identified in this study through systematic reviews and primary nursing research, while bibliometric studies incorporating additional databases could provide a more comprehensive view of the field.

## 1. Introduction

Children with disabilities constitute a substantial global population, with nearly 240 million children estimated to live with some form of disability worldwide [1]. They frequently experience complex and long-term health needs and face persistent inequalities in access to healthcare and health outcomes [2]. Addressing these diverse needs requires comprehensive assessment, care coordination, health promotion, psychosocial support, family-centered care, and continuity of care [3]. Within this continuum, nursing contributes substantially to supporting the health of children with disabilities and their families across healthcare and community settings [4].

Nurses play a central role in meeting the complex needs of children with disabilities and their families across healthcare and community settings [3,4,5]. Their role extends beyond the management of physical health problems to encompass family-centered care, health promotion, psychosocial support, care coordination, and advocacy, thereby contributing to comprehensive and continuous care [3,4,5,6]. Pediatric nursing also includes approaches that strengthen family support and parental self-efficacy, alongside the integration of digital health technologies [7].

The nursing literature on children with disabilities encompasses diverse disability groups, care settings, and areas of practice [4,5,6,7,8]. In such a broad and multidimensional field, understanding where research efforts have been concentrated, which topics have received the greatest research attention, and how collaboration patterns have developed can help researchers identify established areas, knowledge gaps, and future research priorities. Several systematic and scoping reviews have synthesized evidence on specific aspects of nursing care for children with disabilities, such as family-centered care and barriers and facilitators to nursing practice [4,5,6]. However, because these reviews focus on specific topics or care domains, they do not provide a comprehensive understanding of publication trends, major research themes, the temporal development of research topics, or collaboration patterns across the field. Bibliometric analysis can address this gap by providing a quantitative overview of publication trends, major research themes, the temporal development of research topics, and collaboration networks [9,10].

Despite the breadth of nursing research on children with disabilities, a comprehensive bibliometric analysis specifically focused on this body of nursing literature is still lacking. Therefore, this study aimed to provide a comprehensive bibliometric overview of nursing research on children with disabilities by analyzing publication trends, major research themes, the temporal development of research topics, and collaboration patterns.

## 2. Materials and Methods

### 2.1. Data Source and Search Strategy

This bibliometric study was based on data retrieved from the Web of Science Core Collection (WoSCC), a widely used database for bibliometric research that provides standardized bibliographic data suitable for bibliometric and network analyses [9,10]. Using a single data source also ensured consistency in the metadata analyzed across the dataset. The literature search was conducted on 29 June 2026, using the Topic search field. All records and citation information were downloaded on 29 June 2026, and all subsequent analyses were conducted using this fixed dataset.

The following search query was applied: TS = ((disab* OR “developmental delay” OR “intellectual disab*” OR “cerebral palsy” OR “autism spectrum disorder” OR “Down syndrome”) AND (nurs* OR “nursing care” OR “pediatric nurse”) AND (child* OR pediatric* OR infant* OR toddler* OR adolescen*)). No restrictions on publication year were applied during the initial search, yielding 2510 records published between 1977 and 2026.

To assess whether alternative disability-related terms would substantially change the retrieved corpus, additional searches were conducted. Terms containing the disab-stem, such as “developmental disab” and “physical disability,” did not retrieve additional records because they were already captured by the wildcard term disab* in the original search strategy, which covers variants such as disability, disabilities, and disabled. Additional searches using “visual impairment,” “vision impairment,” “vision loss,” “motor impairment,” and “sensory impairment” yielded 59 additional records. Title- and abstract-level assessment showed that 11 (18.6%) of these records were directly focused on children with an established disability, whereas most of the remaining records concerned screening, risk assessment, diagnosis, prevention, or broader clinical conditions. Additional searches were also conducted using the hearing- and vision-related terms “hearing loss,” deaf*, “hearing impairment,” and “blindness.” Examination of the additionally retrieved records showed that many involved screening or assessment in general child populations rather than studies of children with established disabilities. These records commonly addressed newborn or childhood hearing screening, auditory assessment, retinopathy of prematurity screening and prevention, and school-based vision screening. For example, only 8 of the 70 records (11.4%) uniquely retrieved by “hearing impairment” and 5 of the 64 records (7.8%) uniquely retrieved by “blindness” were directly disability-focused. These findings showed that adding alternative disability-related terms increased retrieval but also introduced many studies outside the intended disability-focused scope. Therefore, the original search strategy was retained to preserve the focus of the study.

### 2.2. Eligibility Criteria and Data Collection

The retrieved records were subsequently refined by limiting the document types to Article and Review Article, the citation indexes to the Science Citation Index Expanded (SCI-EXPANDED) and Social Sciences Citation Index (SSCI), and the language to English. After applying these eligibility criteria, 1915 records were included in the final dataset. After these filters were applied, the resulting 1915 records constituted the final dataset; no further exclusion step was performed.

To assess the representativeness of the search strategy, all 1915 records were reviewed across the Web of Science Topic fields, including titles, abstracts, author keywords, and Keywords Plus. Nursing-related terminology was identified in 1911 records (99.8%), indicating a high level of correspondence between the retrieved dataset and the intended nursing-related scope. Only four records (0.2%) reflected incidental matches to the wildcard term nurs*. Consistent with established bibliometric guidance, this verification focused on determining whether the query-defined dataset adequately represented the intended scope of the study rather than applying an additional post hoc classification based on the degree to which nursing constituted the primary focus of each publication [9]. Therefore, the original dataset of 1915 publications was retained for bibliometric analysis.

**Figure 1:**
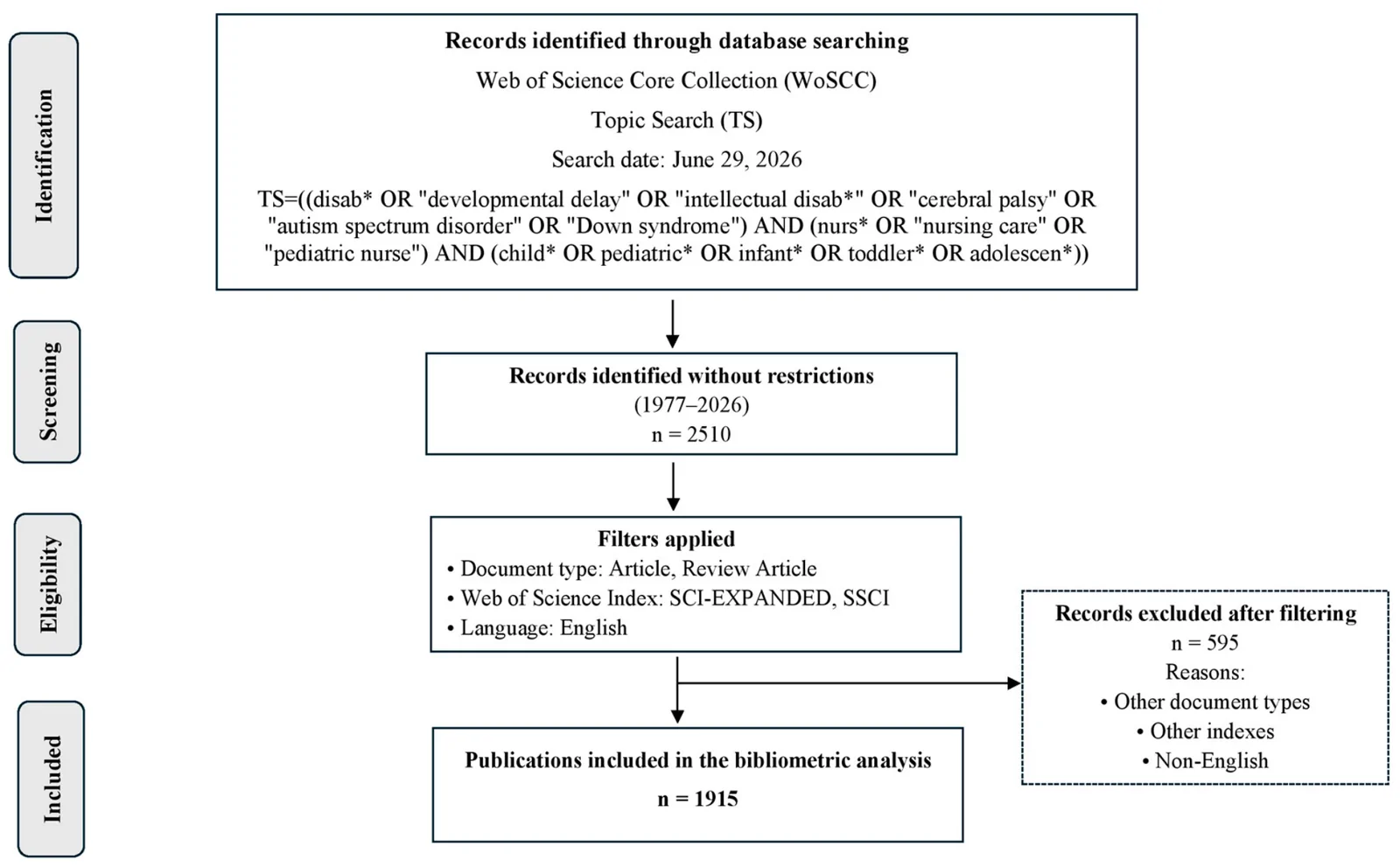
Flowchart of the literature search and selection process. Note. The asterisk (*) denotes a wildcard character used to retrieve all terms sharing the same root (e.g., disab* = disability, disabilities, and disabled; nurs* = nurse, nurses, and nursing; child* = child and children; adolescen* = adolescent and adolescents). SCI-EXPANDED: Science Citation Index Expanded; SSCI: Social Sciences Citation Index.

The eligible records were exported in “Plain Text File” format using the “Full Record and Cited References” record content option. Because Web of Science limits the number of records that can be exported in a single batch, the dataset was exported in batches of 500 and subsequently merged into a single dataset for bibliometric analysis. The study selection process is shown in Figure 1.

### 2.3. Bibliometric Analysis

Bibliometric analyses were performed using VOSviewer (version 1.6.20) [11] and the bibliometrix package (version 5.4.1) through its Biblioshiny web interface in R (version 4.6.1) [12]. Standardized author keywords were used in all keyword-based analyses. Preliminary VOSviewer and Biblioshiny outputs were inspected to identify synonymous or variant keyword terms and inconsistencies in country naming, after which the affected analyses were repeated using standardized data.

VOSviewer was used to construct and visualize bibliometric networks, including author keyword co-occurrence, country co-authorship, and institutional co-authorship networks. Full counting was retained as the primary counting method because, in the present dataset, it provided a more parsimonious and interpretable representation of the network structure. To assess the robustness of the findings, all VOSviewer network analyses were repeated using fractional counting while the dataset, preprocessing procedures, and inclusion thresholds were kept unchanged. Fractional counting resulted in a larger number of clusters and differences in network weights; however, the principal network structures and the most prominent nodes remained broadly consistent across the two counting methods. Therefore, the main network-level findings were not materially affected by the choice between full and fractional counting. The networks were generated using the full counting method and association-strength normalization. Before the keyword co-occurrence analysis, a thesaurus file was applied to standardize author keywords by merging synonymous terms, abbreviations, singular and plural forms, British and American spelling variants, and equivalent expressions. Country-name variants referring to the same country were also standardized before the final country co-authorship analysis. Alternative threshold values were examined for the VOSviewer analyses, and the final thresholds were selected to retain the principal network structure while maintaining adequate interpretability. Across the tested thresholds, the number of included nodes varied, whereas the main network structure and the most prominent nodes remained broadly consistent. A minimum occurrence threshold of 11 was applied in the keyword co-occurrence analysis. Country and institutional collaboration networks were generated using co-authorship analysis, with minimum thresholds of 7 and 10 documents for inclusion, respectively. Total link strength (TLS) was used as an additional quantitative measure of network connectivity in the country and institutional co-authorship analyses [11].

Biblioshiny was used to conduct thematic map, thematic evolution, and trend topic analyses. For consistency across keyword-based analyses, author keywords were standardized using the same principles applied in the VOSviewer analysis, including the merging of synonymous terms, abbreviations, singular and plural forms, British and American spelling variants, and equivalent expressions. The complete list of keyword groups standardized for the Biblioshiny analyses is provided in Supplementary Table S1. The thresholds used in the Biblioshiny analyses were selected based on preliminary parameter testing to balance thematic coverage and interpretability. The thematic map was generated using a co-word analysis approach and the Louvain clustering algorithm. Themes were positioned according to Callon’s centrality and density [13], where centrality reflects a theme’s degree of connection with other themes and density reflects its internal development. The analysis included the 250 most relevant standardized author keywords, with a minimum cluster frequency of 3 per 1000 documents, α = 0.5, community repulsion = 0.5, three labels per cluster, and a label size of 0.3. As a sensitivity analysis, the thematic map was re-run using a higher minimum cluster frequency of 5 per 1000 documents while all other analytical parameters were kept unchanged to assess the stability of the thematic structure across different keyword thresholds. The main thematic clusters were retained across the two thresholds.

To explore the temporal evolution of research themes, thematic evolution analysis was conducted using the Louvain clustering algorithm. The analysis included the 250 most relevant standardized author keywords, a minimum cluster frequency of 5 per 1000 documents, α = 0.5, a minimum weight index of 0.1, three labels per cluster, and a label size of 0.3. Consistent with recent guidance emphasizing that time segmentation in thematic evolution should be logically justified and aligned with the developmental trends of the field [14], the time slices were defined considering the marked increase in publication output over time. Accordingly, the study period was divided into five consecutive time slices with approximately balanced publication volumes rather than equal calendar durations: 1977–2008, 2009–2015, 2016–2019, 2020–2022, and 2023–2026. These periods included 416, 364, 350, 348, and 437 publications, respectively. This approach was used to reduce the potential influence of large differences in publication volume on the thematic evolution analysis. Sensitivity analyses were conducted by varying both the temporal partitioning and the minimum cluster frequency. First, the thematic evolution analysis was repeated using an alternative set of time boundaries (1977–2002, 2003–2010, 2011–2015, 2016–2020, and 2021–2026). Second, the analysis was repeated using a lower minimum cluster frequency of 3 per 1000 documents instead of 5 per 1000 documents. All other analytical parameters were kept unchanged. Across both sensitivity analyses, the overall thematic structure and principal thematic patterns remained broadly consistent, indicating that the main findings were not materially affected by the tested variations in temporal segmentation or minimum cluster frequency.

Finally, trend topic analysis was performed to examine temporal patterns in research topics using standardized author keywords, with a minimum keyword frequency of 5 and a maximum of two keywords displayed per year. As a sensitivity analysis, the analysis was repeated using a lower minimum keyword frequency of 3, while all other parameters were kept unchanged. The overall temporal pattern was broadly comparable across the two thresholds, with the lower threshold primarily introducing additional low-frequency terms.

## 3. Results

### 3.1. Bibliometric Performance Analysis

A total of 2510 records were initially retrieved from the Web of Science Core Collection. Following the application of the predefined eligibility criteria, 1915 publications published between 1977 and 2026 were included in the final bibliometric analysis. The included publications were distributed across 525 source journals and had received a total of 51,846 citations, with an average of 27.06 citations per publication and an H-index of 102 (Table 1).

**Figure 2:**
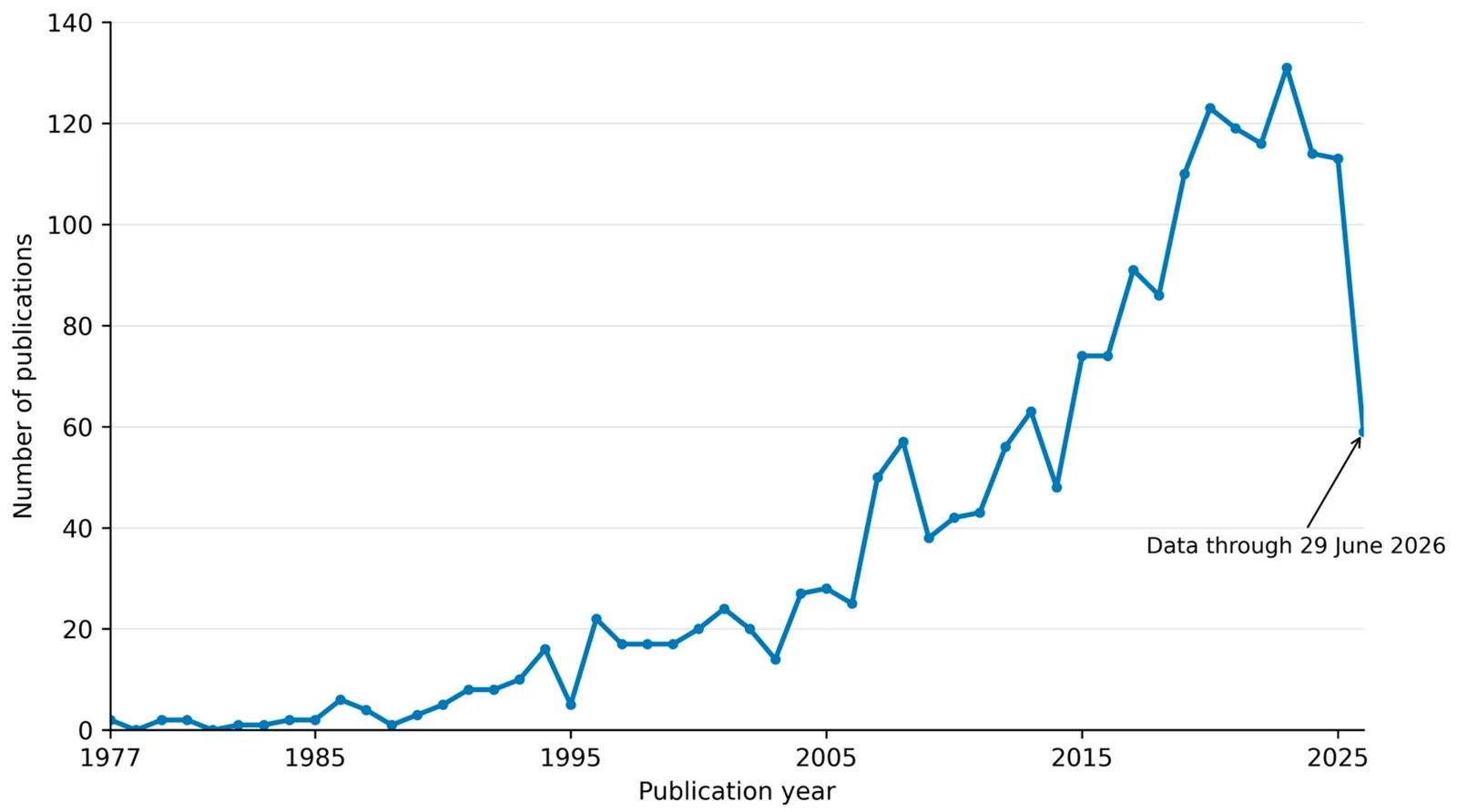
Annual publication trend in nursing research on children with disabilities from 1977 to 2026. Note: data for 2026 include publications indexed up to 29 June 2026.

The annual publication trend demonstrated a steady increase in nursing research on children with disabilities over the study period (Figure 2). Only a small number of publications were identified during the first two decades, whereas publication output increased progressively after 2000 and accelerated markedly during the last decade. Annual publication output exceeded 100 publications from 2019 onward, reaching a peak of 131 publications in 2023. The lower publication count in 2026 reflects the partial coverage of the year, as the analysis included publications indexed up to 29 June 2026.

As presented in Table 2, the Journal of Pediatric Nursing published the highest number of articles (*n* = 81), followed by the Journal of Advanced Nursing and Pediatrics. The United States had the highest number of publications, followed by England, Canada, Australia, and Türkiye. Nursing and Pediatrics were the two most frequently represented research areas, followed by Rehabilitation, Psychology, Public, Environmental and Occupational Health, and Neurosciences and Neurology.

### 3.2. Science Mapping Analysis

**Figure 3:**
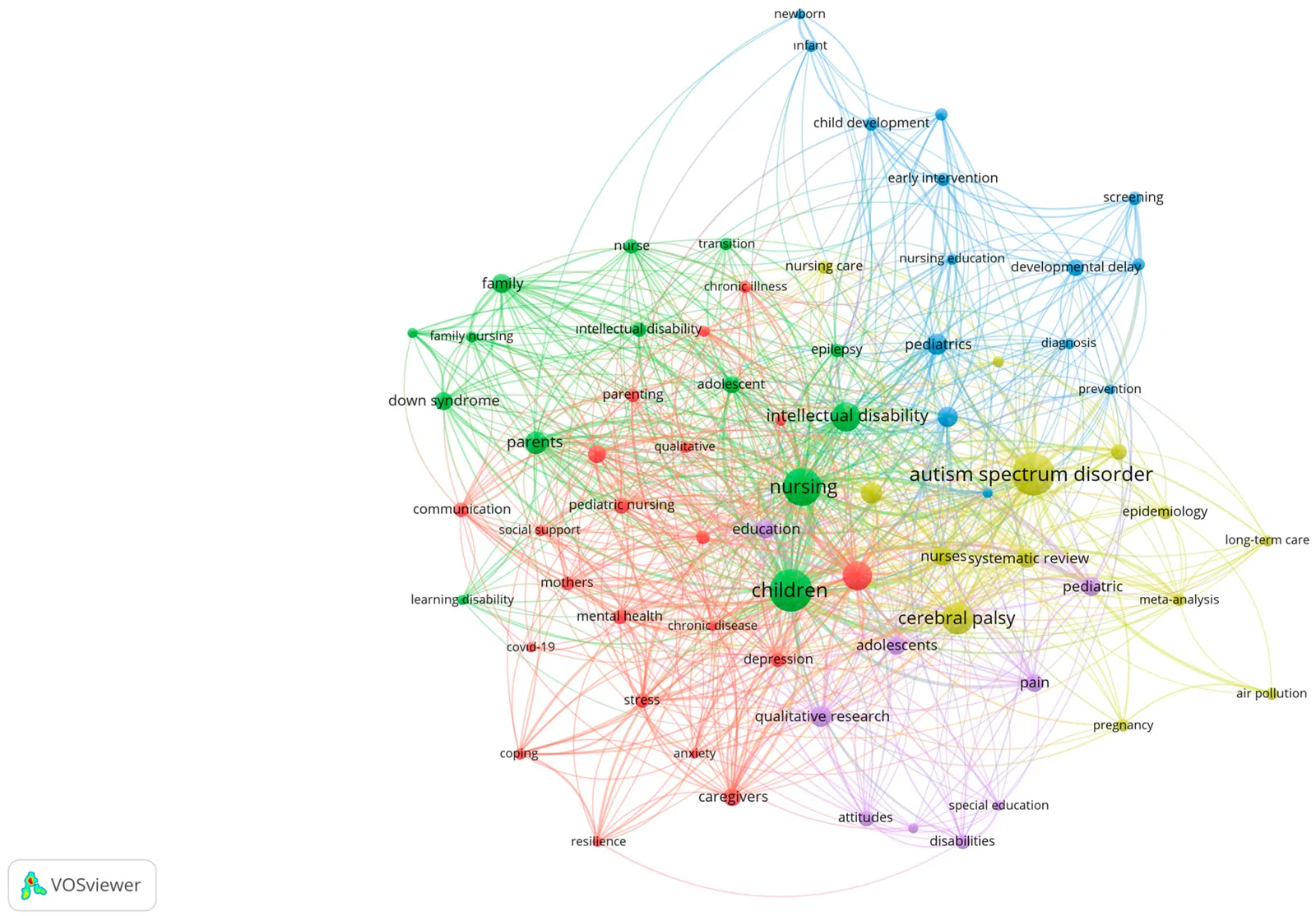
Author keyword co-occurrence network in nursing research on children with disabilities.

After applying the thesaurus file and the predefined occurrence threshold (minimum occurrence = 11), 71 author keywords were included in the co-occurrence analysis and grouped into five thematic clusters (Figure 3). The largest nodes included nursing, children, autism spectrum disorder, intellectual disability, and cerebral palsy, reflecting their relatively high occurrence frequencies within the keyword network. The network also showed close connections among autism spectrum disorder, intellectual disability, cerebral palsy, pediatric nursing, education, mental health, rehabilitation, family, parents, and caregivers.

**Figure 4:**
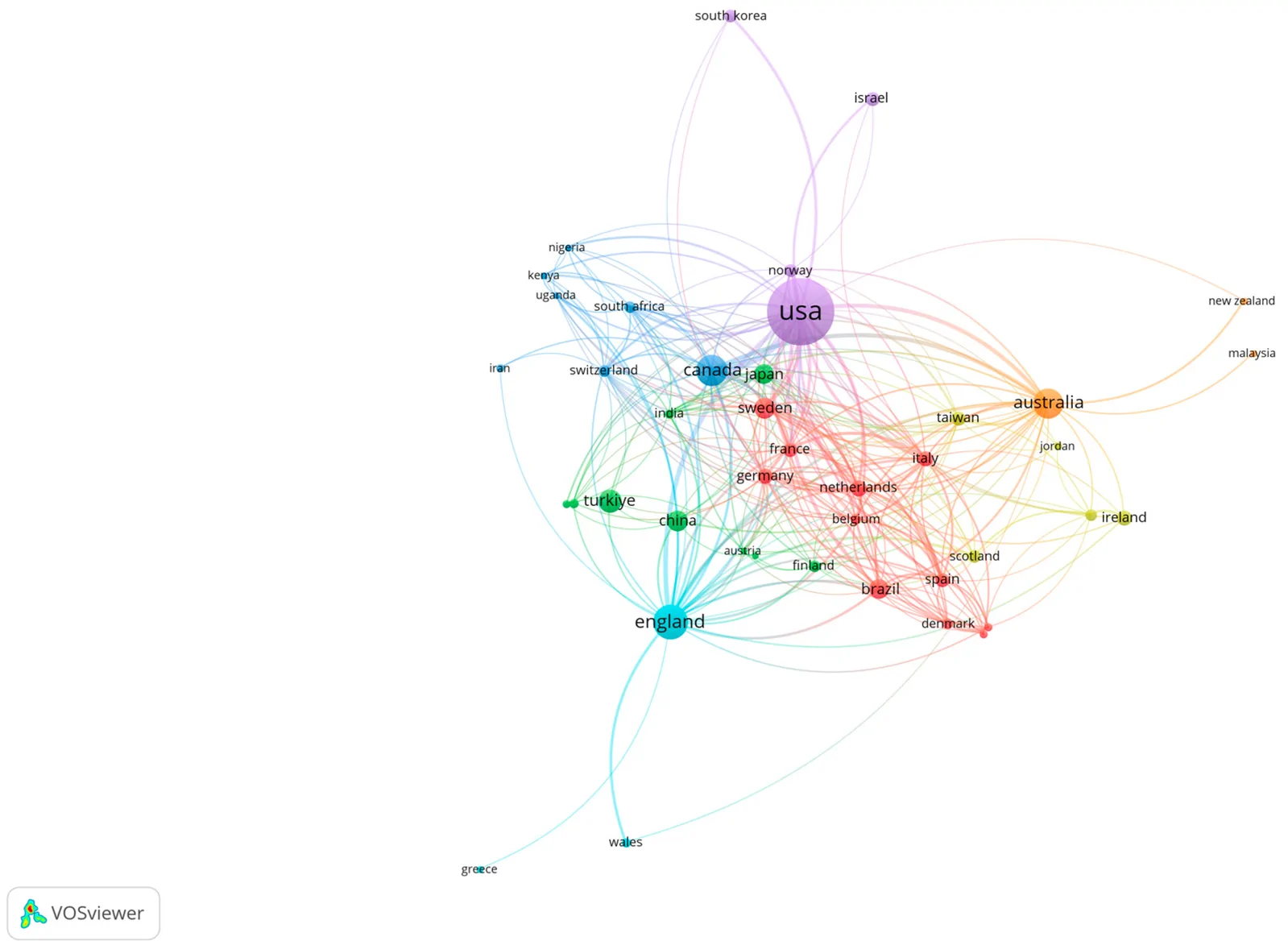
International country collaboration network in nursing research on children with disabilities.

Country co-authorship analysis, including countries with a minimum of seven publications, identified 42 countries participating in the international collaboration network (Figure 4). The United States had the highest total link strength (TLS = 221), followed by England (TLS = 139), Canada (TLS = 114), and Australia (TLS = 97), reflecting their relatively high co-authorship connectivity within the network.

**Figure 5:**
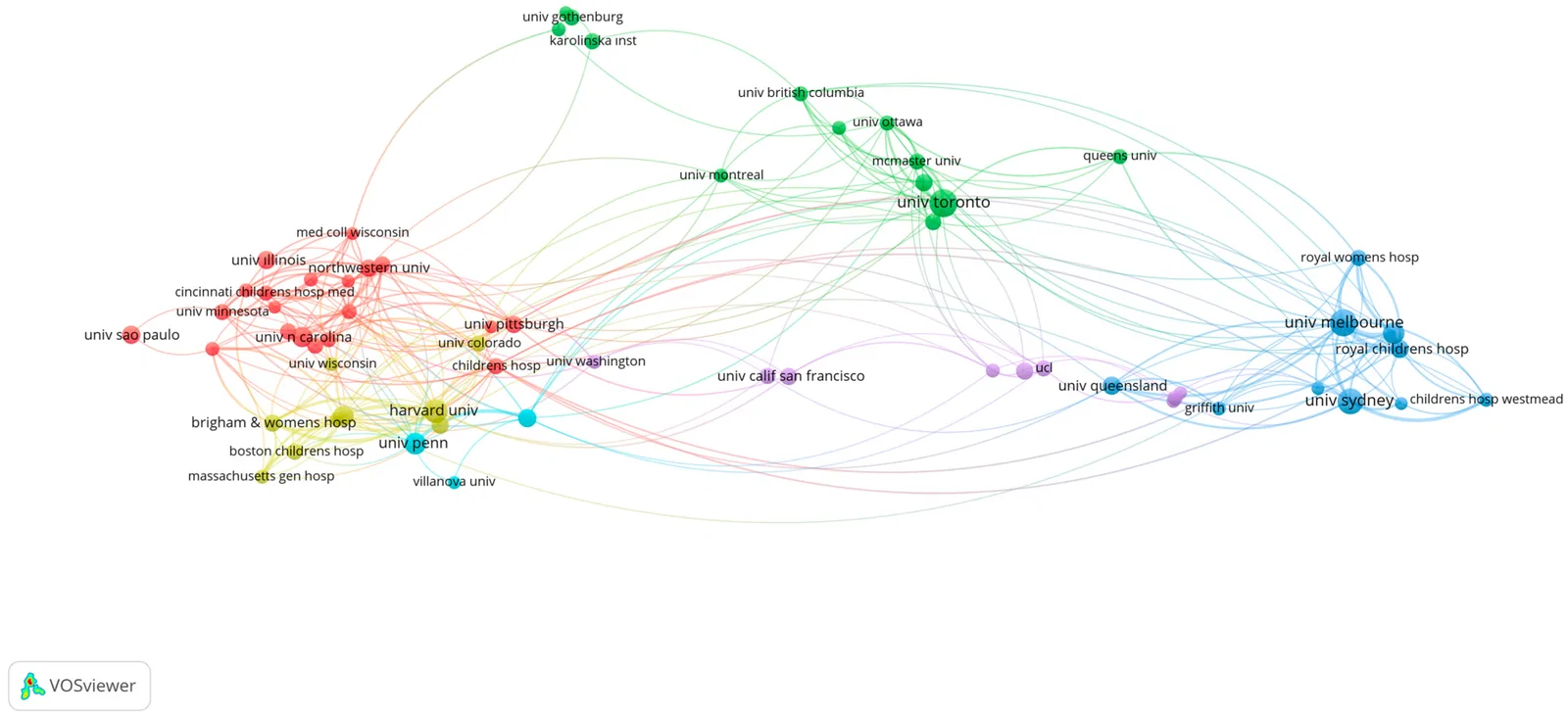
Institutional co-authorship network of nursing research on children with disabilities.

Institutional co-authorship analysis (minimum number of documents = 10) identified 64 institutions organized into six collaboration clusters (Figure 5). The University of Melbourne had the highest total link strength (TLS = 80), followed by the Murdoch Children’s Research Institute (TLS = 63), Harvard University (TLS = 49), the Royal Children’s Hospital (TLS = 48), and the University of Toronto (TLS = 46), reflecting their relatively high co-authorship connectivity within the institutional collaboration network.

**Figure 6:**
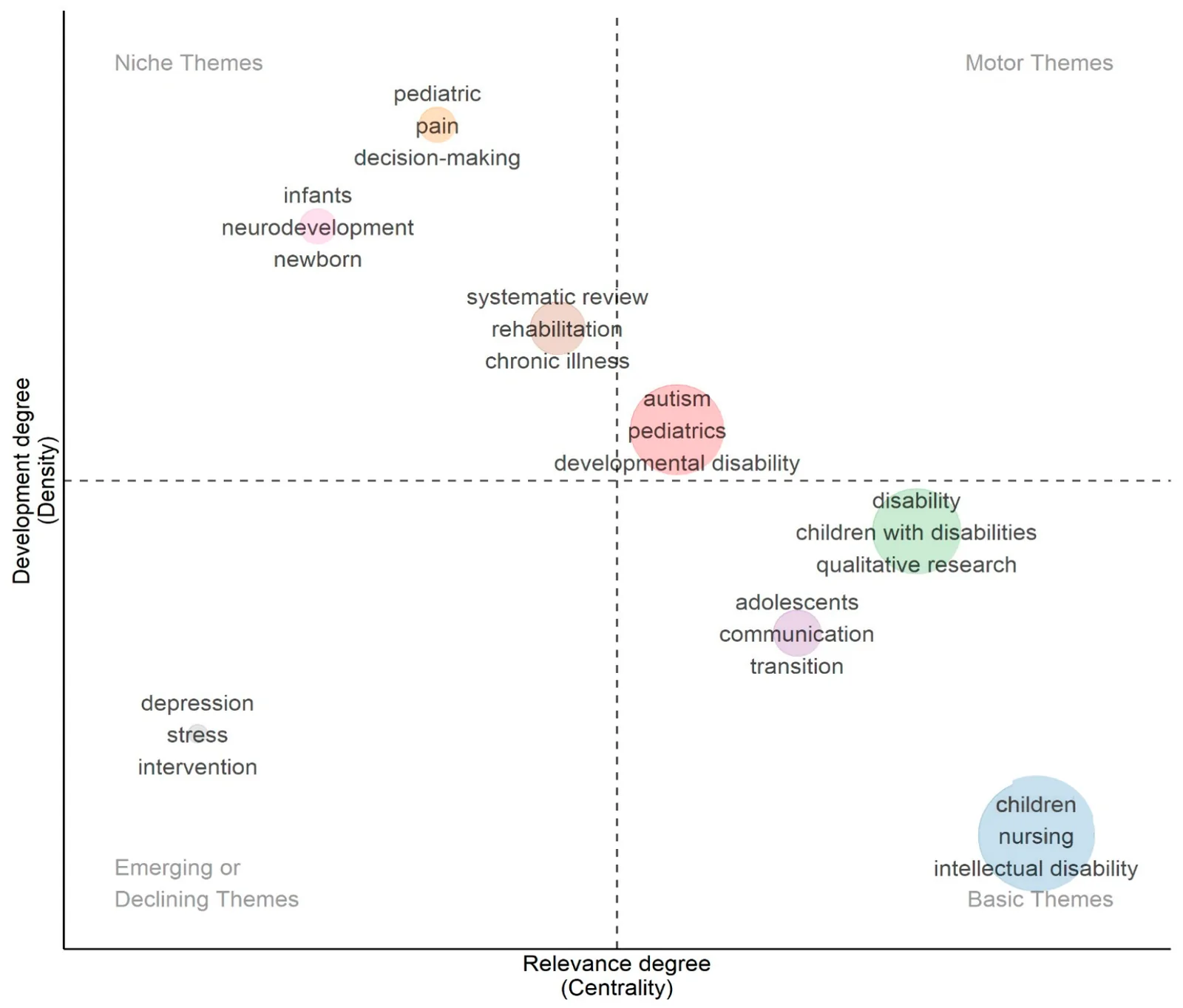
Thematic map of standardized author keywords in nursing research on children with disabilities.

Figure 6 presents the thematic map based on the standardized author keywords according to their centrality and density. The niche themes quadrant included the pediatric–pain–decision-making, infants–neurodevelopment–newborn, and systematic review–rehabilitation–chronic illness clusters. The autism–pediatrics–developmental disability cluster was positioned near the intersection of the centrality and density axes, slightly within the motor themes quadrant. The basic themes quadrant included the disability–children with disabilities–qualitative research, adolescents–communication–transition, and children–nursing–intellectual disability clusters. The depression–stress–intervention cluster was located in the emerging or declining themes quadrant.

**Figure 7:**
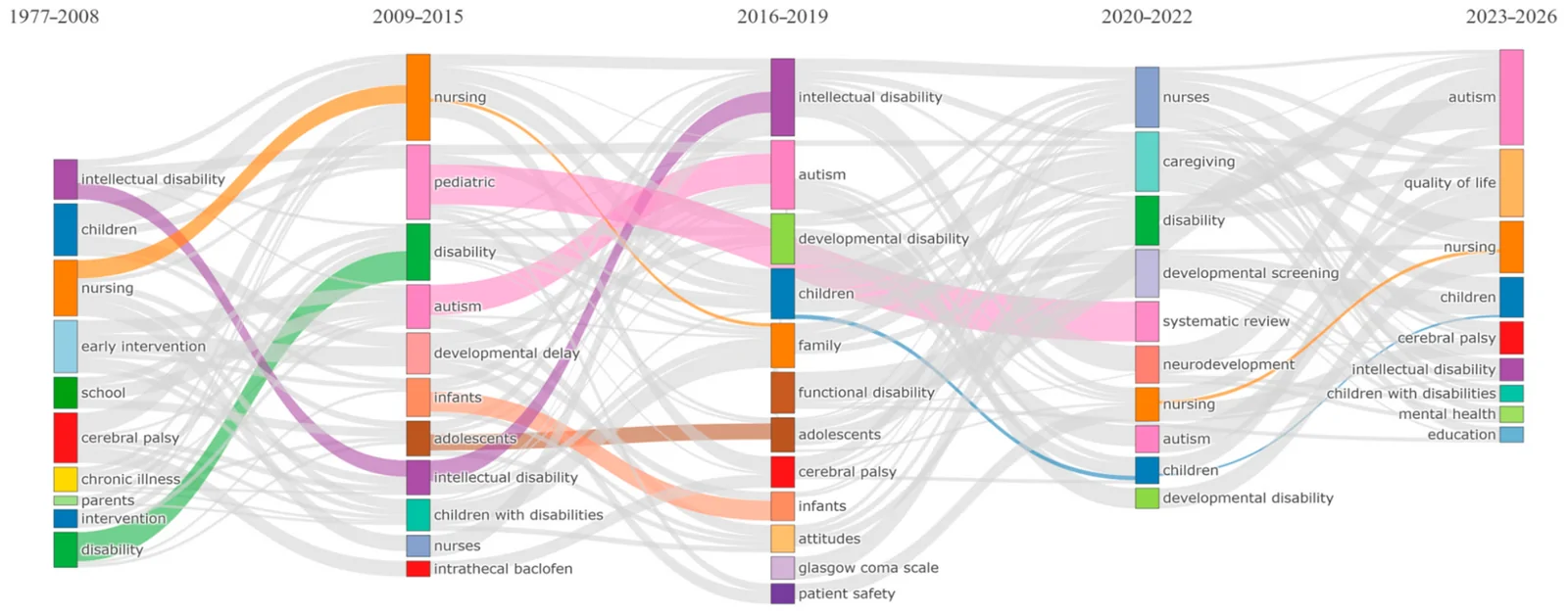
Thematic evolution of standardized author keywords in nursing research on children with disabilities across five time periods (1977–2026).

Figure 7 illustrates the thematic evolution of standardized author keywords across five time periods. In 1977–2008, the themes included school, disability, early intervention, intervention, chronic illness, children, intellectual disability, parents, nursing, and cerebral palsy. In 2009–2015, nursing, pediatric, disability, autism, developmental delay, infants, intellectual disability, adolescents, nurses, children with disabilities, and intrathecal baclofen were represented. In 2016–2019, the themes included intellectual disability, autism, children, developmental disability, functional disability, family, infants, cerebral palsy, adolescents, Glasgow Coma Scale, patient safety, and attitudes. In 2020–2022, nurses, caregiving, disability, developmental screening, systematic review, neurodevelopment, nursing, autism, developmental disability, and children were represented. In 2023–2026, the themes included autism, quality of life, nursing, children, cerebral palsy, intellectual disability, children with disabilities, education, and mental health. Autism, nursing, children, and intellectual disability appeared in four of the five time periods, with only autism present in all four most recent periods. Cerebral palsy and disability appeared in three periods. Among the themes displayed in Figure 7, caregiving, developmental screening, systematic review, and neurodevelopment were first represented in 2020–2022, whereas quality of life, education, and mental health were first represented in 2023–2026.

**Figure 8:**
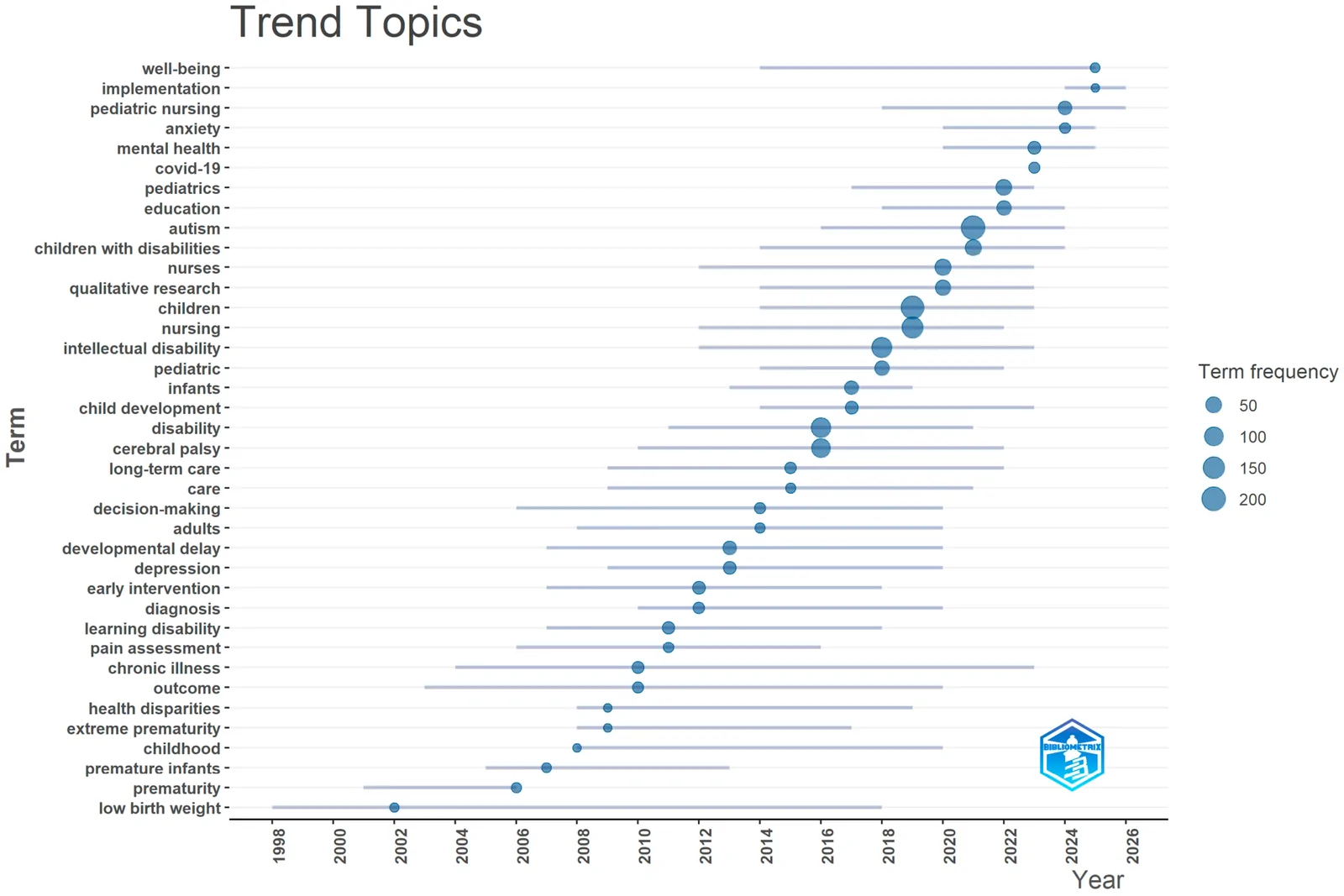
Trend topics based on standardized author keywords over time.

Trend topic analysis showed the temporal distribution of standardized author keywords (Figure 8). Based on the median publication year, earlier topics included low birth weight, prematurity, premature infants, childhood, extreme prematurity, health disparities, chronic illness, outcome, learning disability, and pain assessment. More recent topics included pediatrics, education, mental health, COVID-19, pediatric nursing, anxiety, well-being, and implementation.

## 4. Discussion

Nursing research on children with disabilities increased steadily after 2000, with a sharper rise during the last decade. Similar upward publication trends have been reported in related areas, such as pediatric family-centered care and healthcare services for children with disabilities [15,16]. This increase may reflect the growing recognition of childhood disability as a public health and equity issue, together with increasing emphasis on family-centered care, community-based services, and the long-term support needs of children with disabilities [2,3,6].

Journal of Pediatric Nursing was the leading source of publications in the present study. This finding is consistent with bibliometric studies of pediatric family-centered care and pediatric nursing, in which it was also identified as a leading publication source [15,17]. Other journals that ranked highly in our analysis, including Journal of Advanced Nursing, Pediatrics, Journal of Clinical Nursing, and Child: Care, Health and Development, have also been identified as leading publication sources in related research [15,18]. This distribution shows that nursing research on children with disabilities is published across both nursing and broader pediatric and child health journals.

In the present study, the United States was the leading contributor in terms of publication output in nursing research on children with disabilities, followed by England, Canada, and Australia. Similar geographical patterns have been reported in related bibliometric studies, with the United States and several other high-income countries among the leading contributors [15,16,19]. This pattern is also consistent with global nursing research, where scientific output is concentrated in countries such as the United States, the United Kingdom, Australia, and Canada [20]. This may partly reflect the greater economic capacity and higher levels of development of these countries, as gross domestic product, gross national income, and human development have been associated with nursing research productivity [21].

The keyword co-occurrence analysis showed that nursing research on children with disabilities is centered on major disability groups, particularly autism, intellectual disability, and cerebral palsy, together with themes such as family, mental health, education, and rehabilitation. Similar thematic patterns have been reported in bibliometric studies of disability-related research, with intellectual disability, autism, cerebral palsy, mental health, and rehabilitation emerging as prominent themes [16,22]. This thematic breadth is also reflected in the distribution of research areas in the present study, with nursing and pediatrics predominating alongside rehabilitation, psychology, and public health. The breadth and interconnectedness of these themes highlight the multidisciplinary nature of the field and the importance of collaboration and coordination across disciplines in addressing the complex needs of children with disabilities and their families [23,24].

The country collaboration network showed that the United States occupied a prominent position, with England, Canada, and Australia also showing extensive international connections. Similar patterns have been reported in related research on children with disabilities and in community health nursing, where the United States and other leading countries played prominent roles in international collaboration networks [16,25]. These findings show that international collaboration in this field is already well established, although some countries show stronger international connections within the network. For researchers seeking to expand international collaboration, these networks may help identify countries with more extensive international co-authorship connections. Partnerships with researchers from these countries may facilitate knowledge exchange, access to broader research networks, and the development of more sustained international collaborations. At the same time, extending these partnerships to less represented countries could broaden the geographical diversity of future collaborative research.

The institutional collaboration network revealed several well-connected institutions in Australia and North America, including the University of Melbourne, Murdoch Children’s Research Institute, Harvard University, the Royal Children’s Hospital, and the University of Toronto. A similar clustered institutional collaboration structure has been reported in a bibliometric nursing study, where multiple interconnected institutional groups were identified and institutions within the same clusters tended to collaborate closely [26]. Partnerships between more highly connected and less connected institutions may offer opportunities for sharing expertise, methodological experience, and research resources, and may contribute to broader and more sustained institutional collaboration networks.

The thematic map showed that the autism–pediatrics–developmental disability cluster was positioned near the intersection of the centrality and density axes, slightly within the motor themes quadrant, indicating a relatively well-developed and well-connected thematic structure. Notably, autism spectrum disorders were also positioned among the motor themes in the bibliometric analysis by Gulgosteren et al. [16], showing a similar thematic pattern across the two studies. In contrast, the children–nursing–intellectual disability cluster was identified as a basic theme, indicating relatively high centrality but comparatively lower internal development. Intellectual disability was likewise positioned among the basic themes in the study by Gulgosteren et al. [16]. The niche positioning of the infants–neurodevelopment–newborn cluster suggests a well-developed but more specialized research area with relatively limited connections to other themes.

The thematic evolution analysis showed both continuity and diversification in nursing research on children with disabilities over time. Autism, nursing, children, and intellectual disability were represented across four of the five periods, indicating continuity in the thematic structure of the field. At the same time, later periods included additional care-related, developmental, and psychosocial topics, with quality of life, education, and mental health first represented in the most recent period. A similar broadening has been reported by Gulgosteren et al., who found that research on health services for children with disabilities expanded from themes such as cerebral palsy, children, access, and gross motor function to include greater emphasis on care, diagnosis, and parents [16]. Taken together, these patterns suggest that the field has retained several recurring disability- and nursing-related themes while also showing greater thematic diversification over time.

The trend topic analysis further showed a temporal shift toward more recent nursing-related, psychosocial, and implementation-oriented topics. Well-being, implementation, pediatric nursing, anxiety, and mental health were among the topics with the most recent median publication years, together with COVID-19. The more recent positioning of mental health is consistent with previous bibliometric research showing increasing attention to mental health in the literature on children with disabilities [16]. This psychosocial focus is also reflected in the related caregiver literature. Gull and Kaur identified anxiety, depression, stress, and other psychological concerns as major themes in their bibliometric analysis of caregivers of children with disabilities [27]. Together, these findings indicate an overlap between the recent psychosocial topics identified in the present nursing literature and themes reported in the related caregiver literature.

This study has several limitations. First, although the WoSCC is a widely recognized and well-established source of standardized bibliographic data for bibliometric research, the use of a single database may have resulted in the omission of relevant publications indexed only in other databases. Consequently, the findings should be interpreted as reflecting the literature indexed in WoSCC rather than the entire body of available literature on this topic. Second, the restriction to English-language articles and reviews indexed in SCI-EXPANDED and SSCI was applied to enhance the consistency and comparability of the dataset; however, this approach may have excluded relevant studies published in other languages, document types, or indexes. Third, citation indicators may have been affected by the long publication period covered by the study. Older publications had more time to accumulate citations, while highly cited publications may have disproportionately influenced the average citation count. The H-index may likewise have been influenced by the longer citation accumulation time available to older publications. Fourth, author-keyword availability and indexing practices may have varied over the long study period, particularly in earlier records. Although the time slices were defined to achieve approximately balanced publication volumes and sensitivity analyses were conducted using alternative time boundaries, the observed temporal patterns may still partly reflect differences in metadata availability, publication volume, and the chosen time boundaries rather than genuine changes in nursing research. Finally, because the literature search was conducted on 29 June 2026, data for 2026 reflect only a partial year and should therefore be interpreted accordingly.

## 5. Conclusions

This bibliometric analysis provides a comprehensive overview of nursing research on children with disabilities from 1977 to 2026. Research output increased substantially over time, particularly during the last decade. Across the study period, the United States had the highest publication output and co-authorship connectivity, followed by England, Canada, and Australia. The keyword network showed a broad and interconnected structure, with nursing, children, autism spectrum disorder, intellectual disability, and cerebral palsy among the largest nodes. The thematic map showed that the autism–pediatrics–developmental disability cluster was positioned near the intersection of the centrality and density axes, slightly within the motor themes quadrant, whereas the children–nursing–intellectual disability cluster was positioned among the basic themes. The thematic evolution analysis revealed both continuity and diversification, with autism, nursing, children, and intellectual disability represented across multiple periods, while quality of life, education, and mental health were represented in the most recent period. Trend topic analysis further showed that well-being, implementation, pediatric nursing, anxiety, and mental health were among the topics with more recent median publication years. Overall, the findings indicate an evolving and thematically diverse research field, with recurring disability-specific topics alongside more recent psychosocial, pediatric nursing, and implementation-related topics.

Greater participation of less represented countries and institutions may contribute to broader representation in future collaborative research. The diversity of themes identified across the analyses reflects the multidimensional nature of nursing research on children with disabilities. In this context, multidisciplinary collaboration and effective care coordination across health, rehabilitation, mental health, and community services may be beneficial, with nurses potentially contributing to communication, service coordination, and continuity of care. Future research could build on the thematic patterns identified in this study by examining these areas in greater depth through systematic reviews and primary nursing research, while broader bibliometric studies incorporating additional databases could provide a more comprehensive view of the field.

## Figures and Tables

**Table 1:** Descriptive bibliometric profile of the dataset.

Characteristic	Value
Initial records retrieved	2510
Publications included in the final analysis	1915
Publication period	1977–2026
Number of source journals	525
Total citations	51,846
Average citations per publication	27.06
H-index	102

**Table 2:** Leading journals, countries, and research areas in the included publications.

Journal	*n*	Country	*n*	Research Area	*n* (%)
Journal of Pediatric Nursing	81	United States	743	Nursing	661 (34.5)
Journal of Advanced Nursing	58	England	203	Pediatrics	485 (25.3)
Pediatrics	57	Canada	157	Rehabilitation	161 (8.4)
Journal of Clinical Nursing	44	Australia	154	Psychology	159 (8.3)
Child: Care, Health and Development	36	Türkiye *	89	Public, Environmental & Occupational Health	159 (8.3)
Cochrane Database of Systematic Reviews	29	Sweden	71	Neurosciences & Neurology	154 (8.0)
Journal of School Nursing	23	China	70	General & Internal Medicine	143 (7.5)
Pain Management Nursing	19	Japan	66	Health Care Sciences & Services	125 (6.5)
Developmental Medicine & Child Neurology	18	Brazil	64	Education & Educational Research	115 (6.0)
Journal for Specialists in Pediatric Nursing	18	Netherlands	48	Psychiatry	103 (5.4)

Note: * Publication counts for Türkiye were obtained by combining records indexed under both “Turkey” and “Türkiye” in the Web of Science Core Collection. Because publications in Web of Science may be assigned to more than one research area, the percentages reported for research areas are not expected to sum to 100%.

## Data Availability

The bibliographic data used in this study were retrieved from the Web of Science Core Collection. The processed data supporting the findings of this study are available from the corresponding author upon reasonable request.

## Supplementary Materials

The following supporting information can be downloaded at: https://www.mdpi.com/article/10.3390/healthcare14172824/s1, Table S1: Standardization of author keywords used in the Biblioshiny analyses.

## Abbreviations

The following abbreviations are used in this manuscript:

SCI-EXPANDED	Science Citation Index Expanded
SSCI	Social Sciences Citation Index
TS	Topic (Web of Science search field)
WoSCC	Web of Science Core Collection
