# Supplementary material for: Research Trends and Hot Topics in Nursing Research on Children with Disabilities: A Bibliometric Analysis from 1977 to 2026
Source: Healthcare (Basel). 2026 Sep 3;14(17):2824. doi: 10.3390/healthcare14172824 (PMC13566380; doi:10.3390/healthcare14172824)
Supplement: Supplementary file 1 [file healthcare-14-02824-s001.zip › healthcare-4498157-supplementary.pdf]

Table S1. Standardization of author keywords used in the Biblioshiny analyses

Note. The standardized term is the term retained in the analysis. Merged author-keyword variants are conceptually equivalent forms mapped to the standardized term. Only synonyms, abbreviations, singular/plural forms, British/American spelling variants, and equivalent expressions were merged; conceptually distinct terms were retained separately.

Standardized term (term retained in the analysis)	Merged author-keyword variants (equivalent forms mapped to the standardized term)
autism	autism spectrum disorder; autism spectrum disorders; asd; autism spectrum disorder (asd); autism spectrum disorder - asd; autism spectrum disorders (asd); autistic spectrum disorder; autistic spectrum disorder (asd)
children	child
caregivers	caregiver; carers; carer
family	families
children with disabilities	children with disability; disabled children; disabled child
developmental disability	developmental disabilities
intellectual disability	intellectual disabilities; mental retardation
infants	infant
pediatric	paediatric
pediatrics	paediatrics
attitudes	attitude
disability	disabilities
learning disability	learning disabilities; learning disabil-ities
adolescents	adolescent
parents	parent
nurses	nurse
mothers	mother
transition	transitions
intervention	interventions
chronic illness	chronic illnesses
chronic disease	chronic diseases
young adults	young adult
hospital	hospitals
outcome	outcomes
school nurses	school nurse
risk factors	risk factor
preterm infants	preterm infant
physical disability	physical disabilities
nursing homes	nursing home
experiences	experience
nursing students	nursing student
fathers	father
nurse practitioners	nurse practitioner
students	student
adults	adult
cognitive impairment	cognitive impairments
premature infants	premature infant
pediatric nursing	paediatric nursing
pediatric nurse	pediatric nurses; paediatric nurse; paediatric nurses

Standardized term (term retained in the analysis)	Merged author-keyword variants (equivalent forms mapped to the standardized term)
pediatric otolaryngology	paediatric otolaryngology
pediatric epilepsy	paediatric epilepsy
family-centered care	family-centred care; family centred care; family -centered care
decision-making	decision making
healthcare	health care
home care	homecare
child care	childcare
preschool children	pre-school children
preschool	pre school
health care transition	healthcare transition
low birth weight	low birthweight
health-related quality of life	health related quality of life
children with special health care needs	children with special healthcare needs
healthcare workers	health care workers
healthcare professionals	health care professionals
grounded theory	groundedtheory
